# The Fundus Red Reflex Test Using a Smartphone Showed High Accuracy Compared to an Autorefractor to Detect Refractive Errors in Children

**DOI:** 10.18502/jovr.v21.18164

**Published:** 2026-08-06

**Authors:** Muhammad Syauqie, Lutfah Rif'ati, Wienta Diarsvitri, Sutanto Priyo Hastono, Kemal Nazaruddin Siregar, Nila Djuwita Farieda Moeloek

**Affiliations:** ^1^Department of Ophthalmology, Faculty of Medicine, Andalas University, Padang, Indonesia; ^2^Center for Preclinical and Clinical Medical Research, National Research and Innovation Agency, Jakarta, Indonesia; ^3^Department of Community Medicine, Faculty of Medicine, Hang Tuah University, Surabaya, Indonesia; ^4^Department of Biostatistics and Population Studies, Faculty of Public Health, Universitas Indonesia, Depok, Indonesia; ^5^Department of Ophthalmology, Faculty of Medicine, University of Indonesia / Cipto Mangunkusumo Hospital, Jakarta, Indonesia

**Keywords:** Autorefractor, Children, Refractive Errors, Sensitivity, Smartphone-based Application, Specificity

## Abstract

**Purpose:**

To determine the accuracy of the fundus red reflex test using a smartphone and autorefractor to detect refractive errors in children.

**Methods:**

This cross-sectional study included 1302 eyes of 651 school-age children aged 6-13 years with visual acuity worse than 6/9 in at least one eye, examined using the Snellen chart. Study participants were selected using purposive sampling, after which non-cycloplegic refractive measurement was performed using two instruments: a smartphone-based application (MD EyeCare) and an autorefractor. The detection of refractive errors using the online platform was compared with autorefraction as the reference standard.

**Results:**

Our study found that 46.10% of participants with abnormal red reflex had significant myopia, 41.20% had nonsignificant refractive errors, and 12.70% had significant hypermetropia. The mean difference between the smartphone-based application and the autorefractor was ≤- 3.00 D (range: - 2.88 to - 2.63), with a sensitivity of 0.89 to 0.90 and a specificity of 0.86 to 0.87 for significant myopia, and the area under the curve was between 0.94 and 0.97. The mean difference between the smartphone-based application and the autorefractor was ≥ +1.00 D (range: +0.13 to +0.38), with a sensitivity of 0.76 and a specificity of 0.97 for significant hypermetropia, and the area under the curve was between 0.88 and 0.96.

**Conclusion:**

A smartphone-based application could be used as a screening tool for refractive errors in children, especially in resource-limited settings.

##  INTRODUCTION

Refractive error, where the eye's shape prevents light from focusing directly on the retina and thus leads to blurred vision, is prevalent among children worldwide, with varying rates across different regions. The prevalence of refractive errors in children was 6.0% (95% CI, 5 to 7%) in Ethiopia,^[1]^ 8.0% (95% CI, 7.4 to 8.1%) in India,^[2]^ 15.9% (95% CI, 14.7 to 17.3%) in Bandung, Indonesia,^[3]^ 31.6% (95% CI, 2.7 to 4.1%) in Kazakhstan,^[4]^ and 20-61% in Los Angeles, USA.^[5]^ Common refractive errors such as myopia, hyperopia, and astigmatism are leading causes of vision impairment in children. Early detection is crucial, as uncorrected refractive errors (URE) can significantly impact a child's quality of life, decrease self-esteem, and affect academic performance and social development.^[6]^ The prevalence of URE in school-age children ranged from 2.26 per 1000 in Southeast Asia to 5.85 per 1000 in America.^[7,8,9]^

Early detection of refractive errors in children is crucial for supporting proper visual development and preventing long-term vision issues. Traditional methods for detecting refractive errors include cycloplegic refraction and retinoscopy, which—while accurate—can be time-consuming and require specialized equipment and trained personnel. Recent advancements have introduced alternative screening tools aimed at improving accessibility and efficiency. The fundus red reflex test, traditionally used to detect opacities in the visual axis, has been adapted for refractive error screening. This test involves observing the reflection of light rays from the retina, which can reveal differences in refractive status between the eyes.^[10,11]^ The MD EyeCare (EyeCare SpA, Chile) is a mobile application designed to detect red reflex in the eyes. This application deactivates the pre-flash feature, which has a function to prevent red reflex in photography. The MD EyeCare allows modern smartphones such as iPhones to capture the eye red reflex.^[12]^ Recent studies have explored smartphones to capture these reflexes, offering a portable and efficient screening method.^[13,14,15]^

Despite advances in visual screening technology, a resource-efficient, high-sensitivity, and user-friendly visual screening tool is needed. This research aimed to determine the accuracy of the red reflex test using a smartphone application and an autorefractor to detect refractive errors in children.

##  METHODS 

### Study Design and Samples

This cross-sectional study was conducted on 651 children (1302 eyes) in an elementary school in Padang, Indonesia. The sample size was calculated using a formula for diagnostic test accuracy based on sensitivity.^[16]^ The desired sensitivity was set at 91.0%. The prevalence of refractive errors in children used in the sample size formula was 12.1%.^[3]^ Participants were selected using purposive sampling. The inclusion criteria were students aged 8-13 years with visual acuity worse than 6/9 in at least one eye, examined using the Snellen chart. Students with refractive media abnormalities (including cornea, aqueous humor, lens, or vitreous) or palpebral abnormalities that hinder visualization of the refractive media (e.g., ptosis, tumor), infectious and inflammatory diseases of the eyeball, and eye surgery, in addition to uncooperative participants during the examination, were excluded from the study. We only excluded one child in this study due to congenital ptosis. Almost all children who failed screening were included in this study using a consecutive sampling method. The information related to the study was explained beforehand, and consent was obtained from the parents. The study protocol has been reviewed and approved by the Ethics Committee of the Faculty of Public Health, Universitas Indonesia (Ket-541/UN2.F10.D11/PPM.00.02/2024).

### Data Collection

All children underwent visual acuity screening using the 6/9 line of the tumbling E chart. Children who failed screening underwent visual acuity examination with the Snellen chart, performed by trained non-specialist examiners. An ophthalmologist examined the external structures and refractive media of both eyes in all children using a penlight and an ophthalmoscope. Ocular photographs of each child's eyes were taken using the MD EyeCare application installed on an iPhone 6s (Apple Inc., Cupertino, California, USA) to obtain fundus red reflex images.^[12]^ The images were captured in a dimly lit room (3-10 lux).^[16]^ To measure environmental lighting, we used an AS803 digital lux meter. The light source brightness was adjusted so that the light intensity fell within the desired lux value.

The working distance between the smartphone camera and the child's eye was 40 —60 cm,^[17]^ and the photographs were taken off-axis. The off-axis approach required children to fixate at an angle of 15º from the horizontal meridian.^[17]^

Off-axis photorefraction was performed using an iPhone 6s to capture the eye images. All photographs taken by non-specialists and the ophthalmologist were input into an online platform to compare the results. The detection of refractive errors using the online platform was compared with the performance of the autorefractor as the reference standard. The measurements included red reflex distribution, width, dioptric power, sensitivity, and specificity.

Objective refraction was evaluated under non-cycloplegic conditions by an ophthalmologist using an autorefractor (Tonoref II, Nidek Co., Japan) as the reference standard. Autorefractor readings were obtained three times for each eye, and the results were averaged. The dioptric power was defined as the spherical equivalent, which was calculated by adding the sum of the sphere power with half of the cylinder power.

### Statistical Analysis

The red reflex width and dioptric power were not normally distributed. The correlation between dioptric power and red reflex distribution and width was analyzed using the Spearman correlation test. The receiver operating characteristics (ROC) curves were analyzed to find the area under the curve (AUC), sphericity, spherical equivalent refraction, sensitivity, and specificity.^[18]^ Analyses were conducted using SPSS version 26.0 (IBM Corp., Armonk, New York, USA).

##  RESULTS

Table 1 shows that of 651 children aged 6-13 years old (Mean [SD]: 9.31 [1.98]; 95% CI, 9.09-9.53), 52.10% were males and 47.90% were females. The most common red reflex finding was significant myopia (46.10%), followed by nonsignificant refractive errors (41.20%) and significant hypermetropia (12.70%).

**Table 1:** Characteristics of study participants and red reflex distribution

**Characteristics**	**Frequency**	**Percentage**
Sex		
Male	339	52.10
Female	312	47.90
Red reflex distribution		
Significant hypermetropia	83	12.70
Significant myopia	300	46.10
Nonsignificant refractive errors	268	41.20
Note: Participants were 6-13 years old.		

Table 2 shows significant correlations between refractive power (diopters) and red reflex width (*P* < 0.001). In addition, mean values and 95% CI are presented for significant hypermetropia, significant myopia, and nonsignificant refractive errors.

**Table 2:** Correlation between dioptric power and red reflex width

	**Mean**	**95% CI**	***P*** -value ${}^{*}$
Pupil size	5.81	5.70 to 5.91	
Red reflex width			< 0.001
Significant hypermetropia (mm)	2.63	2.42 to 2.84	
Significant myopia (mm)	1.94	1.86 to 2.02	
Nonsignificant refractive errors (mm)	5.59	5.50 to 5.68	
Dioptric power			< 0.001
Significant hypermetropia (D)	1.36	0.94 to 1.78	
Significant myopia (D)	–5.98	–6.39 to –5.56	
Nonsignificant refractive errors (D)	–1.17	–1.33 to –1.02	
${}^{*}$ Spearman correlation.			

**Figure 1:**
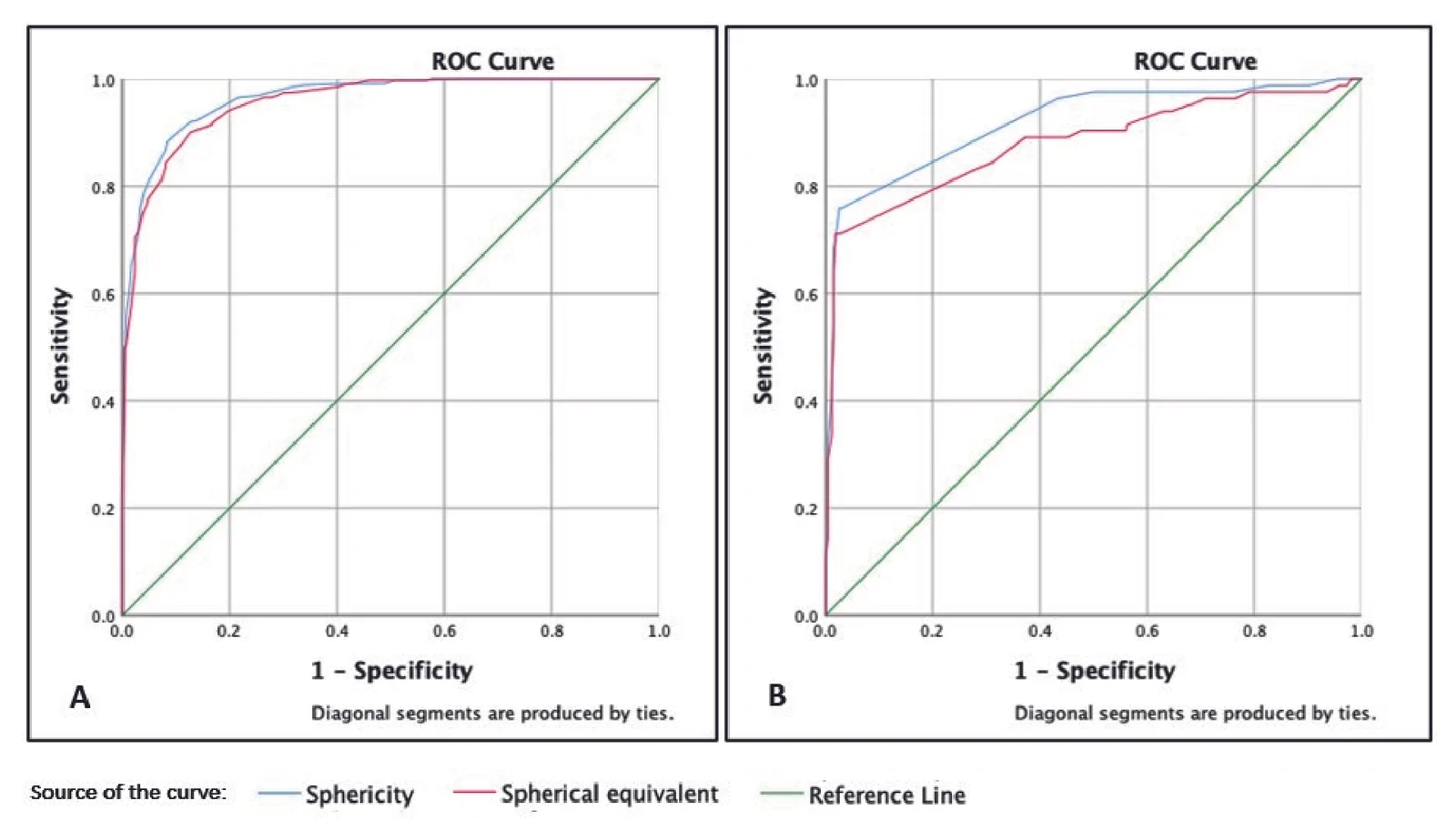
ROC curve for dioptric power in (A) significant myopia and nonsignificant refractive errors (*P* < 0.001) and (B) significant hypermetropia and nonsignificant refractive errors (*P* < 0.001).

Figure 1A shows the ROC curve for dioptric power in significant myopia and nonsignificant refractive errors. The AUC for sphericity was 0.96 (95% CI: 0.94 to 0.97, *P* < 0.001), and for spherical equivalent refraction was 0.95 (95% CI: 0.93 to 0.96, *P* < 0.001). Figure 1B shows the ROC curve for dioptric power in significant hypermetropia and nonsignificant refractive errors. The AUC for sphericity was 0.92 (95% CI: 0.88 to 0.96, *P* < 0.001), and for spherical equivalent refraction was 0.88 (95% CI: 0.83 to 0.93, *P* < 0.001).

**Figure 2:**
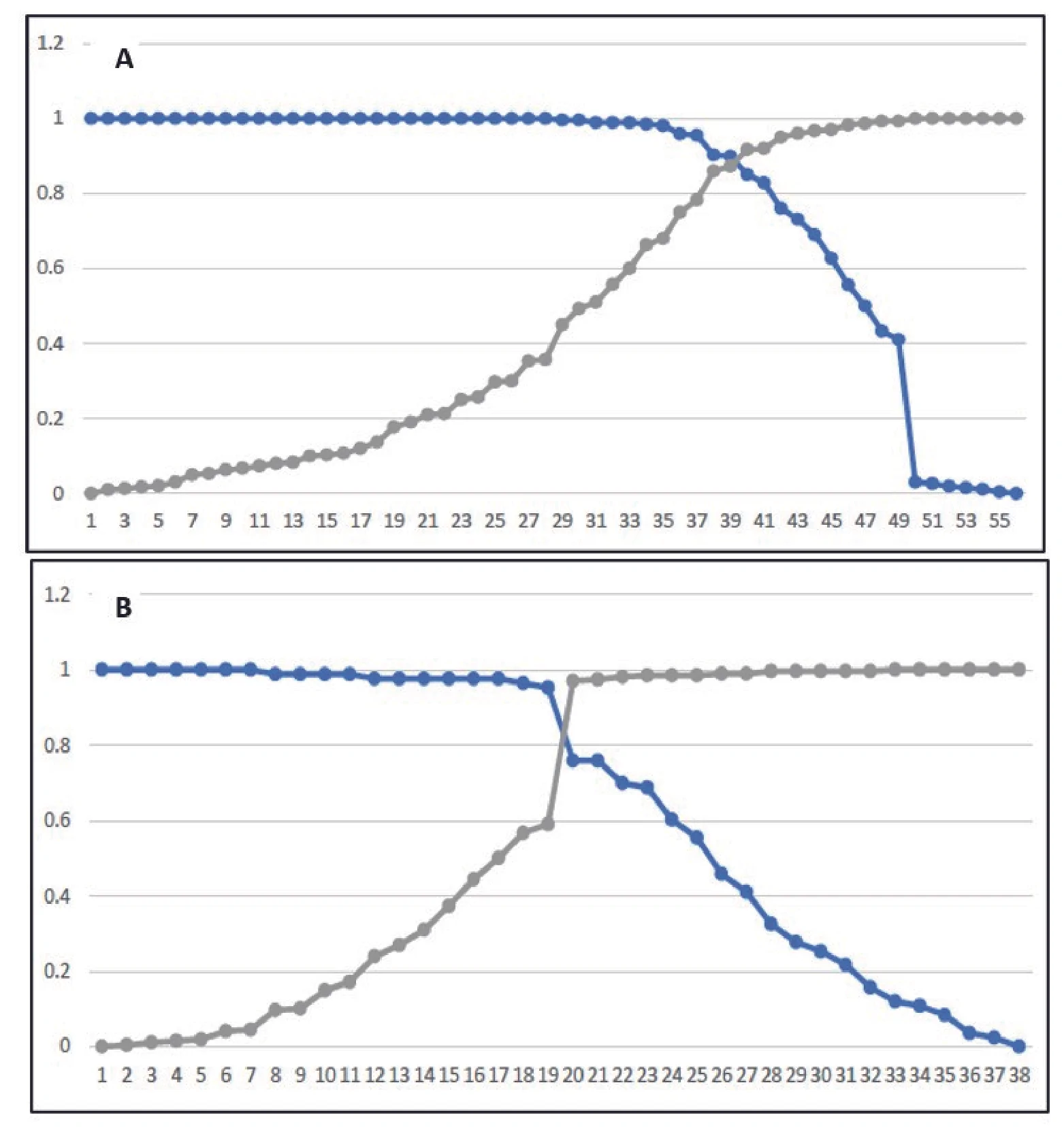
Sensitivity and specificity curve by cut-off values of dioptric power in (A) significant myopia and nonsignificant refractive errors and (B) significant hypermetropia and nonsignificant refractive errors. Blue, sensitivity; gray, specificity.

Figure 2A shows the intersection of the sensitivity and specificity curves for dioptric power in significant myopia and nonsignificant refractive errors. Accordingly, the mean difference between the smartphone-based application and the autorefractor was ≤- 3.00 D (range: - 2.88 to - 2.63), with a sensitivity of 0.89 to 0.90 and a specificity of 0.86 to 0.87, while the AUC was between 0.94 and 0.97. Based on the sensitivity and specificity analysis of dioptric power in significant myopia and nonsignificant refractive errors [Supplementary Material, Appendix 1], optimal sensitivity and specificity values were obtained at dioptric power values of - 2.88 to - 2.63. Based on the red reflex distribution according to dioptric power [Supplementary Material, Appendix 2], a value ≤- 3.00 D was consistently referred to as significant myopia, and a value >- 3.00 D as nonsignificant refractive errors.

Figure 2B shows the intersection of the sensitivity and specificity curves for dioptric power in significant hypermetropia and nonsignificant refractive errors. It indicates the mean difference between the smartphone-based application and the autorefractor was ≥ +1.00 D (range: 0.13 to 0.38), with a sensitivity of 0.76 and a specificity of 0.97, while the AUC was between 0.88 and 0.96. According to the sensitivity and specificity tables for dioptric power in significant hypermetropia and nonsignificant refractive errors [Supplementary Material, Appendix 3], dioptric power values between 0.13 and 0.38 have the optimal sensitivity and specificity. According to the distribution of red reflex values by dioptric power [Supplementary Material, Appendix 2], a value of ≥ +1.00 D consistently corresponded to significant hypermetropia, and a value of < +1.00 D consistently correlated with nonsignificant refractive errors.

**Figure 3:**
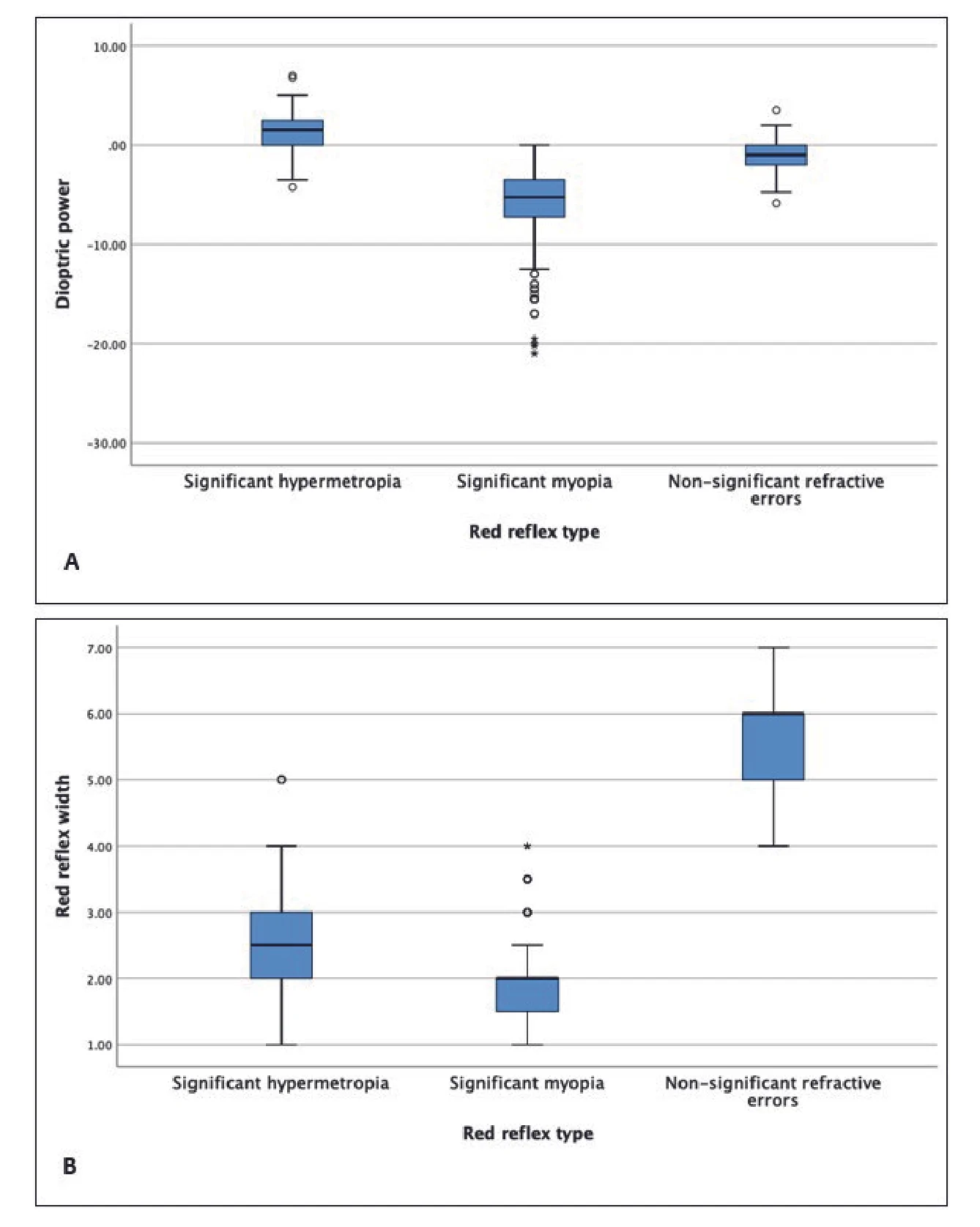
Box plots of (A) red reflex type and dioptric power and (B) red reflex type and red reflex width.

Figure 3 presents the box plots indicating the relationship between red reflex type and dioptric power and between red reflex type and red reflex width.

##  DISCUSSION

Our study found that most children who failed the screening examination presented with significant myopia and hyperopia. This study used total purposive sampling to recruit all children with visual impairment due to refractive error. All elementary school children who failed the screening were included as the study sample. Meanwhile, random sampling would have included children with normal vision, thereby reducing the number of children with visual impairments available for clinical evaluation. The findings are consistent with previously published studies.^[2,3,7]^ Interactions among genetic and environmental factors, delayed diagnosis, and delayed treatment may lead to URE in children. Recent studies reported that children with younger age, low-income conditions, limited resources, remote geographical location, limited outdoor play, and prolonged screen time exposure have an increased risk of delayed detection and treatment for refractive errors.^[19,20,21,22]^

We utilized MD EyeCare to screen refractive errors in children. It is a smartphone-based application designed to detect various eye disorders in children. The application employs AI algorithms to analyze photographs taken with an iPhone camera and uses the photorefractive principle to detect refractive errors through crescent-shaped red reflex identification. While looking upward at a 15º angle, the child's gaze is not aligned directly with the smartphone camera axis, thereby reducing accommodation in young children because of the relatively near working distance. Accommodation induces pupil constriction, which can prevent the appearance of a red reflex. Looking directly at the smartphone camera could also affect the examination result. The off-axis approach avoids the coincidence of the eye's far point with the camera's working distance, especially in myopic eyes, which can affect the instrument's accuracy.^[23]^ In children with myopia, the far point of the eye may coincide with the working distance, which can lead to an underestimation of dioptric power and, consequently, an emmetropic appearance. This can reduce the accuracy of the screening tools.^[23]^ A previous study showed that this approach can optimally capture the eye red reflex using a smartphone camera.^[17]^

The fundus red reflex test is done using off-axis photorefraction to identify crescent-shaped red reflexes, which are direct reflections of the light circle image on the patient's posterior pole, seen at the pupillary plane. They can only be seen with noncoaxial eccentric illumination. The off-axis photorefraction allows this directly reflected image to leak into the camera, forming the crescent-shaped red reflex. With a fixed working distance and light source eccentricity (distance of the light source from the edge of the camera aperture), quantitative measurement of refractive error is achieved by measuring the crescent and pupil size.^[23]^

The results of the smart application were compared to those obtained by autorefractors, established instruments used to measure refractive errors objectively.^[24]^ The accuracy of autorefraction in children exhibited high sensitivity and specificity for detecting hyperopia (84.6% sensitivity, 96.7% specificity) and myopia, especially under cycloplegic conditions.^[25]^

In our study, the AUC was 0.95 for significant myopia and 0.92 for significant hypermetropia. Achieving high AUC values approaching 1.0 is essential for early detection and intervention of refractive errors, which can lead to amblyopia if left uncorrected.^[26]^ Further, our study found that the mean difference between the smartphone-based application and the autorefractor was ≤- 3.00 D (range: - 2.88 to - 2.63), with a high sensitivity of 0.89 to 0.90 and a specificity of 0.86 to 0.87 for significant myopia [Supplementary Material, Appendix 1]. The mean difference between the smartphone-based application and the autorefractor was ≥ +1.00 D (range: 0.13 to 0.38), with a sensitivity of 0.76 and a high specificity of 0.97 for significant hypermetropia [Supplementary Material, Appendix 3]. The cut-off values chosen were also considered clinically, as dioptric power is directly relevant to clinical decision-making. Optimal cut-offs aligned with clinical thresholds, as outlined in Supplementary Material Appendix 2, provide additional guidance for the practical implementation of the results. The dioptric power beyond the cut-off values indicated refractive errors, which cause significantly poor vision in children. Wearing spectacles is expected to improve their vision dramatically, which will increase their compliance with spectacle wear and reduce the false positive referral rate to optometrists or ophthalmologists.

Previous studies^[27,28]^ have shown that examination with autorefractor has a tendency toward minus overcorrection results when used under noncycloplegic conditions, with a mean difference of –1.15 D and –1.23 D, respectively, compared to cycloplegic autorefraction on primary school children regardless of their refractive error status.^[29,30]^ Other previous studies reported a high undercorrection between +1.53 D and +2.06 D, respectively, in children with hypermetropia.^[27]^ This discrepancy could potentially lead to false positive results in children with moderate to high myopia and false negative results in children with moderate to high hypermetropia. Given the discrepancy between cycloplegic and non-cycloplegic autorefraction, any myopia <- 2.50 D and hypermetropia > +1.00 D without cycloplegic instillation need to be detected and managed promptly.^[31]^

Two studies reported results similar to our study. A cross-sectional study of refractive errors was conducted on 292 adolescents (584 eyes) aged 13-19 years in Nigeria. The study population was older than that in our study, with a mean age of 16.6 years. This study used three instruments: a Netra smartphone-based autorefractor, a tabletop autorefractor, and a retinoscope under both non-cycloplegic and cycloplegic conditions. In comparison to our study, this study reported greater variability in refractive errors of ≥ 0.50 D, including myopia, hypermetropia, myopic astigmatism, and hypermetropic astigmatism. This observation was under both cycloplegic and non-cycloplegic conditions using both smartphone-based and tabletop autorefractors, with comparable proportions. The study reported that the smartphone-based autorefractor was as effective as the tabletop autorefractor in detecting refractive errors. Still, it did not perform as well as retinoscopy in either condition.^[32]^ Another study was conducted on 141 school children aged 7-14 years in China. This study measured refractive errors of +0.50 D to - 12.00 D using smartphone cameras and auto-refraction under cycloplegic and non-cycloplegic conditions. In contrast to our study, the smartphone was rotated to four angles: vertical (90º), oblique (45º or 135º), and horizontal (180º). The study indicated that smartphone photorefraction had a high correlation with autorefraction under cycloplegic (*r* = 0.88) and non-cycloplegic (*r* = 0.80) conditions. Both methods yielded a high sensitivity of > 97% in detecting myopia of >- 1.50 D. However, the smartphone photorefraction under non-cycloplegic conditions achieved higher specificity (78%) than its counterpart (72%).^[33]^

We did not perform cycloplegic refraction, which was one of the limitations of this study and could result in underestimation of hypermetropia.^[34]^ However, most young children are mildly hypermetropic and can reach emmetropization thanks to their strong accommodative capacity. Those children with mild levels of hypermetropia rarely develop amblyopia and/or strabismus. Moreover, American Association for Pediatric Ophthalmology and Strabismus (AAPOS) guidelines state that hypermetropia of > +3.50 D in children over 48 months, measured with an automated vision screening instrument, is a refractive error risk factor for amblyopia.^[35]^ From a public health perspective, hypermetropia of > +1.00 is considered significant and warrants spectacle correction in children.^[31]^ The administration of cycloplegic agents for vision screening is limited due to their adverse effects, such as blurred vision, headaches, dry eyes, dry mouth, tachycardia, and arrhythmia.^[35]^ The adverse effects of cycloplegic agents can induce resistance from parents and teachers, which prevents vision screening in the communities. As our study aims to assess the validity of the vision screening instrument in the communities, non-cycloplegic refraction is sufficient to detect significant hypermetropia, which is a risk factor for visual impairment in children.

Another limitation is that this was a single-center study with a mostly Malay population, as it was initially planned as a pilot study. The homogeneous nature of the participants and the study's purposive sampling approach restrict the applicability of findings to asymptomatic populations and may not fully represent children with normal vision. Considering these preliminary promising results, we may expand this research to conduct a multicenter study in the future. In addition, this study used the MD EyeCare application for screening, which cannot be used on Android devices, hence limiting its applicability for smartphone users. Future studies should explore cross-platform applications to improve accessibility in diverse settings, as current findings are limited to iOS-only environments.

In summary, this study is the first to evaluate refractive error screening in school-age children by comparing a smartphone-based application (MD EyeCare) with an autorefractor. The results indicated high sensitivity and specificity for detecting significant myopia, and good sensitivity and high specificity for detecting significant hypermetropia. The smartphone-based application showed potential as a screening tool for refractive errors in children, especially in resource-limited settings.

##  Acknowledgements

The author would like to thank Lembaga Penelitian dan Pengabdian Kepada Masyarakat (LPPM) Universitas Andalas and Lembaga Pengelola Dana Pendidikan (LPDP) Indonesia for research funding under grant No. PD2592024110600195.

##  Financial Support and Sponsorship

None.

##  Conflicts of Interest

None.
